# Radiocarbon analysis reveals expanded diet breadth associates with the invasion of a predatory ant

**DOI:** 10.1038/s41598-017-15105-1

**Published:** 2017-11-03

**Authors:** Wataru Suehiro, Fujio Hyodo, Hiroshi O. Tanaka, Chihiro Himuro, Tomoyuki Yokoi, Shigeto Dobata, Benoit Guénard, Robert R. Dunn, Edward L. Vargo, Kazuki Tsuji, Kenji Matsuura

**Affiliations:** 1 Laboratory of Insect Ecology, Graduate School of Agriculture, Kyoto University, Kitashirakawa-Oiwakecho, Sakyo-ku, Kyoto, 606-8502 Japan; 2 Research Core for Interdisciplinary Sciences, Okayama University, 3-1-1, Tsushimanaka, Okayama, 700-8530 Japan; 3 Ryukyu Sankei Co., Ltd/Okinawa Prefectural Plant Protection Center, Naha, Okinawa, 902-0072 Japan; 4 Laboratory of Conservation Ecology, Faculty of Life and Environmental Sciences, University of Tsukuba, Tsukuba City, Japan; 5 School of Biological Sciences, University of Hong Kong, Pok Fu Lam, Hong Kong; 6 Department of Applied Ecology, North Carolina State University, Raleigh, NC 27695-7617 USA; 7 Department of Entomology, 2143 TAMU, Texas A&M University, College Station, TX 77843-2143 USA; 8 Faculty of Agriculture, University of the Ryukyus, Nishihara, Okinawa, 903-0213 Japan

## Abstract

Invasions are ecologically destructive and can threaten biodiversity. Trophic flexibility has been proposed as a mechanism facilitating invasion, with more flexible species better able to invade. The termite hunting needle ant *Brachyponera chinensis* was introduced from East Asia to the United States where it disrupts native ecosystems. We show that *B. chinensis* has expanded dietary breadth without shifting trophic position in its introduced range. Transect sampling of ants and termites revealed a negative correlation between the abundance of *B. chinensis* and the abundance of other ants in introduced populations, but this pattern was not as strong in the native range. Both termite and *B. chinensis* abundance were higher in the introduced range than in native range. Radiocarbon (^14^C) analysis revealed that *B. chinensis* has significantly younger ‘diet age’, the time lag between carbon fixation by photosynthesis and its use by the consumer, in the introduced range than in the native range, while stable isotope analyses showed no change. These results suggest that in the introduced range *B. chinensis* remains a termite predator but also feeds on other consumer invertebrates with younger diet ages such as herbivorous insects. Radiocarbon analysis allowed us to elucidate cryptic dietary change associated with invasion success.

## Introduction

Tens of thousands of species have been introduced, either intentionally or inadvertently, to locations outside their native ranges^1^. A subset of these species become established in the new locations as invasive species where they have negative impacts on native communities and ecosystems. These invasive species are now recognized as the second most important cause of species extinctions^2^. Species introductions have been the subject of an enormous body of research with thousands of published articles. However, why some species cause such impacts and others do not remains enigmatic.

Ants represent a particularly interesting taxonomic group for studying biological invasions mechanisms as they count among the most ecologically and economically important groups of biological invaders, with five species on the list of the 100 most problematic invaders^3^. Invasive ants often become highly abundant in their introduced range where they reduce native ant diversity and affect other organisms both directly and indirectly as has been well-documented for argentine ants^4,5^, fire ants^6^, crazy ants^7–9^, and Asian needle ants^10^ among others. The ecological effects of invasive ants in introduced ranges depend on a complex interaction of behavioural, ecological and genetic factors, each independently increasing the numerical abundance of the introduced species relative to native species^11^. Recently, a number of studies have highlighted the possibility of species that exhibit behavioral or ecological flexibility may be particularly likely to have strong effects on local communities^12–14^.

In particular, invasiveness may be facilitated by the ability of ants to shift their diets during invasion, whether to consume a wider breath of food items or even, potentially, to consume their competitors. Dietary shifts in introduced populations have been reported in the Argentine ant (*Linepithema humile*) and in the red imported fire ant (*Solenopsis invicta*), both considered among the world’s most destructive introduced species^13,14^. These omnivorous ants shifted trophic positions when they were introduced; in their invasive ranges both species shifted to diets with a greater dependence on honeydew-producing Hemiptera^13,14^. In the case of *S. invicta*, intense competition from other native ants, including *L. humile*, limits access to honeydew resources in its native range^14^, but it is able to dominate these resources in its introduced range in the U.S. It has been proposed that the access to carbohydrates provided by hemipterans contributes to competitive performance of these ants and enhances their ecological success in introduced ranges^14,15^, possibly through increased colony growth associated with greater honeydew consumption^14^. Although the importance of dietary flexibility in invasion success has been studied in hemipteran mutualist ants, nothing is known about potential dietary changes in ants less able to rely on honeydew.

**Figure 1:**
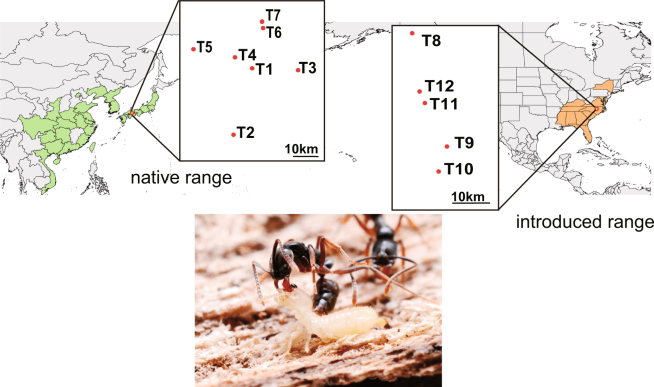
Map displaying the locations of study sites in the native (Japan) and introduced (United States) ranges of the Asian needle ant *Brachyponera chinensis*. The sampling design consisted of twelve 900-m line transects, seven in Japan (T1-T7) and five in the United States (T7-T12). Maps were created with the QGIS software (version 2.18; http://www.qgis.org/en/site/forusers/download.html).

The Asian needle ant, *Brachyponera* *chinensis* (formerly *Pachycondyla chinensis*), widely distributed from Far Eastern Asia to Southeast Asia, was introduced into the southeastern United States in the early 19th century^16^ (Fig. 1). It has since become an abundant invasive species, even within relatively undisturbed forests. The occurrence and increased abundance of *B. chinensis* diminishes the abundance and diversity of native ants^10,17^. Moreover, *B. chinensis* disrupts ant-seed dispersal mutualisms by displacing native ant species, especially the keystone mutualist *Aphaenogaster* species^17,18^. Unlike the Argentine ant and the red imported fire ant, *B. chinensis* does not rely on hemipteran-mutualist providing carbohydrates but is instead a predator of termites in the genus *Reticulitermes* in both its native^19^ and invaded^20^ ranges. It has been suggested that the availability of termite prey in the southeastern United States, combined with the absence of specialist predators of termites, might have facilitated the success of *B. chinensis* ^10,20,21^.

An important limitation in this area of research concerns the absence of observational data of ant diets. To investigate dietary shifts in invasions of hemipteran-mutualist ants, earlier studies assessed how their trophic positions differ between native and introduced populations by comparing stable nitrogen isotope data (δ^15^N). However, the same approach may not be applicable to *B. chinensis* because this species does not tend honeydew excreting hemipterans and thus dietary changes of this predatory invasive ant may occur without shifts in trophic position. An alternative approach is to use other environmentally acquired elements to compare the composition of prey in native and introduced ranges. The use of the natural abundance of radiocarbon (^14^C) makes it possible to explore feeding habits of terrestrial consumers, which could not be revealed by more conventional analyses^22–24^. The testing of nuclear bombs after World War II doubled ^14^C concentration of atmospheric CO_2_. Concentrations have been, predictably, decreasing since the nuclear ban treaty in 1963^25^. The steady decline of atmospheric ^14^CO_2_ with time makes it possible to estimate the ‘diet age’ of a consumer, which is defined as the average time lag between carbon fixation by photosynthesis and its use by the consumer, with an accuracy of 1–2 years^26^. Earlier studies showed a strong association between diet ages of consumers and their known feeding habits^23,26–29^. For example, wood-feeding termites that feed on wood have old diet ages (ca. 10 to > 50 years old). The diet ages of litter-feeding invertebrates (earthworms and termites) range from 3 to 6 years^24,28^. Ant species that consume honeydew and nectar (*Polyrhachis* sp.) have a younger diet age (just one year) than strictly predatory ants (e.g., *Leptogenys diminuta*) (6 years)^24^.

To examine dietary shifts of *B. chinensis* in its introduced range, we conducted stable carbon (^13^C) and nitrogen (^15^N) isotope analyses of termites and ants in both the native and introduced ranges of *B. chinensis*. We also measured diet ages of *B. chinensis* by using radiocarbon ^14^C. We found that *B. chinensis* has markedly changed its prey composition and now relies less on termites in introduced range and more on other non-decomposer invertebrates.

## Results and Discussion

**Figure 2:**
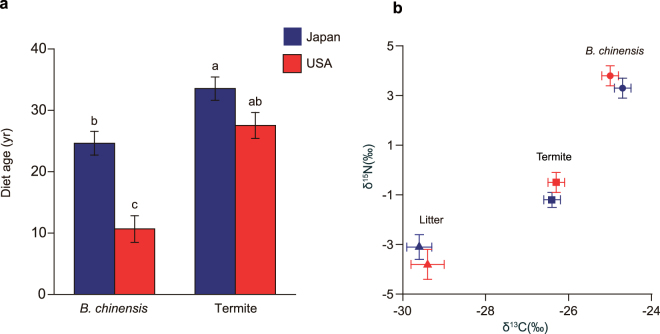
Diet ages and trophic ecology of *B. chinensis* in the native range (Japan) and in the introduced range (United States). (**a**) Diet ages (least square means ± SE) of *B. chinensis* and termites in the native range (Japan) and in the introduced range (United States). Both the study sites and the sample types had significant effects on diet ages of *B. chinensis* and termites (*F* = 14.19, *P* = 0.0037 and *F* = 133.61, *P* < 0.0001, respectively). Interaction effect of native/introduced and sample type was also significant (*F* = 12.79, *P* = 0.0008), where the diet age of *B. chinensis* in the introduced range was significantly younger than that of *B. chinensis* in the native range, but there was no significant difference in the diet ages of termites. Different letters on the bars indicate significant differences among groups (*P* < 0.05, Tukey-Kramer test). (**b**) There was no significant difference between the native range and the introduced range in δ^13^C (least square means ± SE) (*F* = 0.0150, *P* = 0.904) and in δ^15^N (least square means ± SE) (*F* = 0.1809, *P* = 0.678), there was a while highly significant difference among sample types in δ^13^C (*F* = 218.47, *P* < 0.0001) and in δ^15^N (*F* = 287.68, *P* < 0.0001). The interaction term had no significant effect on δ^13^C (*F* = 1.81, *P* = 0.170) or δ^15^N (*F* = 1.94, *P* = 0.149).

Diet ages of termites are associated with feeding habits: termites that inhabit and consume large-diameter wood have older diet ages than litter-feeding and soil-feeding species^24^. As expected, our carbon (^14^C) analysis demonstrated that wood-feeding *Reticulitermes* termites have old diet ages both in Japan (23–48 years, N = 18) and in the Unites States (17–36 years, N = 15), and did not differ between these regions (Fig. 2a; Dataset S1). The diet age of *B. chinensis* in both its native range (18–35 years, N = 14) and in its introduced range (3–18 years, N = 13) were much older than the oldest diet age of the ants reported in previous studies, i.e., *Aenictus* and *Leptogenys* genera (4–6 years)^24^. The extremely old diet ages of *B. chinensis* are in line with the hypothesis that *B. chinensis* relies on termites as prey^10,19–21^, particularly in its native range. However, our comparison of *B. chinensis* in its native and introduced range showed that the diet age of *B. chinensis* in the introduced range was significantly younger than that in the native range (Fig. 2a). We hypothesize, in light of these results, that the Asian needle ant has expanded its diet after invasion and now feeds on prey items that have younger diet ages than wood-feeding termites, most likely other invertebrates such as litter-feeding detritivores and herbivores.

Within a typical ecological community δ^13^C and δ^15^N values are higher in animals with more carnivorous diets, and lower in those that are herbivores (e.g.^30–32^). Earlier studies on trophic ecology of invasive ants showed that the Argentine ant *L. humile* and the red imported fire ant *S. invicta* increased reliance on plant-based resources and thus shifted to lower trophic positions after invasion^13,14^. In contrast to these cases, our stable isotope analysis demonstrated that there was no significant difference in δ^15^N and δ^13^C values of litter (native: N_1_ = 7, introduced: N_2_ = 5), termites (N_1_ = 23, N_2_ = 28) and *B. chinensis* (N_1_ = 21, N_2_ = 25) between the native and introduced range (Fig. 2b; Dataset S2), indicating no significant change in trophic position following its invasion; the ants are still highly predatory. Unlike *L. humile* and *S. invicta*, the Asian needle ant does not consume plant-based carbohydrates provided by honeydew-producing Hemiptera (Fig. 2b). However, even without shifting its trophic position in its introduced range *B. chinensis* appears to acquire carbon from younger sources in its introduced range than in its native range (Fig. 2a). This pattern suggests that native populations of *B. chinensis* are more dependent on prey of old diet age, i.e., termites, and introduced populations consume more prey of younger diet age such as herbivores and litter-feeding invertebrates. Although earlier studies illuminated the importance of exploitation of plant-based resources in ant invasions, our results suggest that increased dietary flexibility within the same trophic level might also facilitate large impacts.

**Figure 3:**
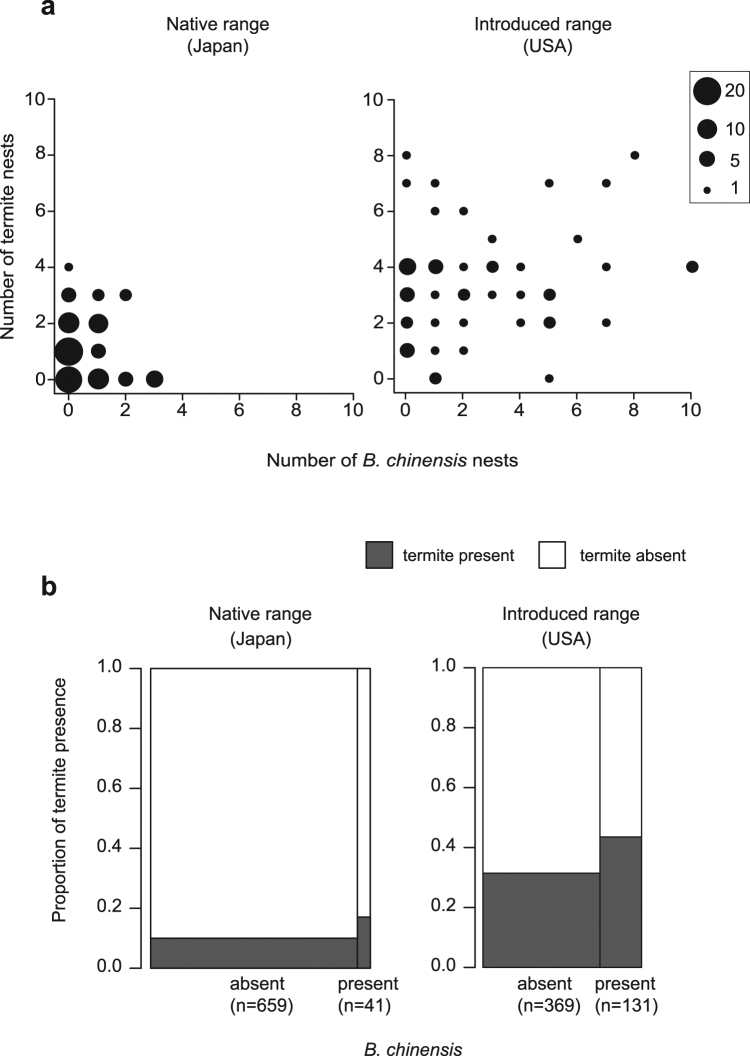
The abundance of *B. chinensis* and termite nests collected at each sampling point. (**a**) Relationship between the number of *B. chinensis* nests and the abundance of termites in the native and introduced ranges of *B. chinensis*. We collected 73 *R. speratus* nests in Japan and 121 *R. flavipes*, 51 *R. virginicus* and one *R. hageni* nests in the United States. There was no significant relationship between the number of *B. chinensis* nests and the abundance of termites in Japan (*χ* ^2^ = 1.01, *P* = 0.32) and in the United States (*χ* ^2^ = 2.58, *P* = 0.11; likelihood ratio test). The size of each plot is proportional to the number of data points it represents. (**b**) Comparison of termite presence between the downed logs with and without *B. chinensis* colonies. The proportion of logs containing termites was higher than those without *B. chinensis* both in the native (*χ* ^2^ = 4.126, *P* < 0.05) and introduced ranges (*χ* ^2^ = 5.243, *P* < 0.05; likelihood ratio test).

There was no significant relationship between the number of *B. chinensis* nests and the abundance of termites in Japan (*χ* ^2^ = 1.01, *P* = 0.32; Table S1) or in the United States (*χ* ^2^ = 2.58, *P* = 0.11; likelihood ratio test; Table S2, Fig. 3a). Both termite nest density and *B. chinensis* nest density were higher in the introduced range than in native range (termite nest: *χ* ^2^ = 15.64, *P* < 0.001; *B. chinensis* nest: *χ* ^2^ = 7.74, *P* < 0.01, likelihood ratio test). The probability of termite presence in logs with *B. chinensis* was significantly higher than in those without *B. chinensis* both in the native (*χ* ^2^ = 4.126, *P* < 0.05) and introduced ranges (*χ* ^2^ = 5.243, *P* < 0.05; likelihood ratio test; Fig. 3b). Colony size of *Reticulitermes* termites is between tens of thousands to hundreds of thousands of individuals^33^, while nest size of *B. chinensis* ranges from 100 to several thousands of individuals^34^. As long as *B. chinensis* colonies live with termite colonies in the same piece of wood, termites are an abundant, high quality, renewable food supply, in many ways similar to the hemipteran honeydew exploited by most other invasive ant species.

**Figure 4:**
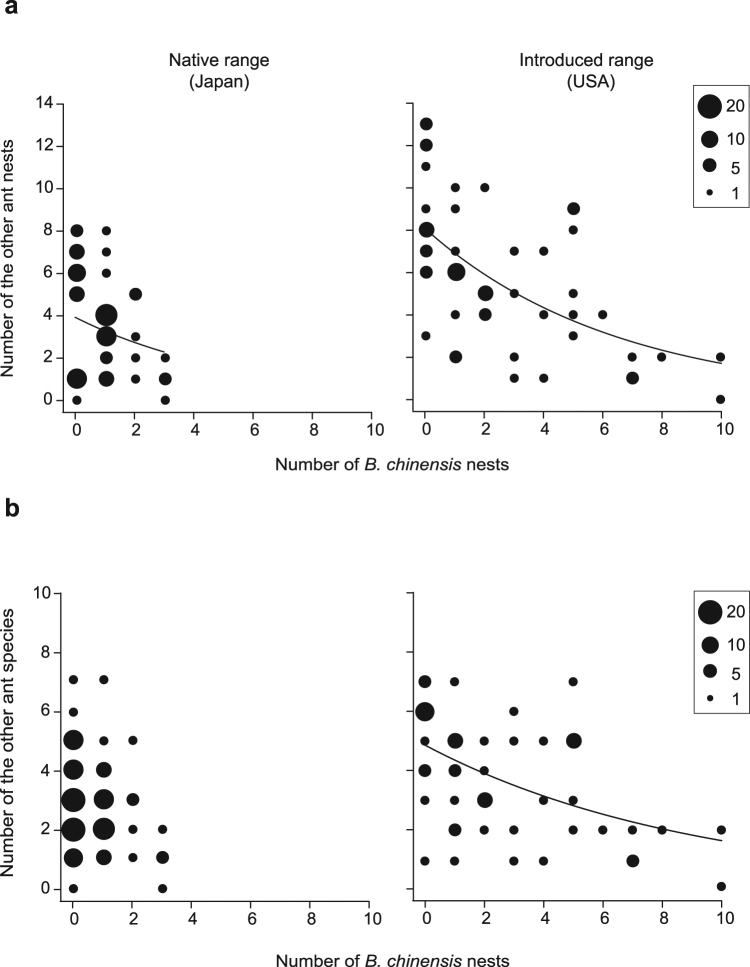
Effect of *B. chinensis* on other ant species. (**a**) There was a significant negative relationship between the number of *B. chinensis* nests and the number of nests of other ants found at the same point (*χ* ^2^ = 29.68, *P* < 0.0001), while marginally non-significant in Japan (*χ* ^2^ = 3.84, *P* = 0.05; likelihood ratio test). (**b**) There was a significant negative relationship between the number of *B. chinensis* nests and the number of the other ant species found at the same point (*χ* ^2^ = 11.99, *P* < 0.001), while there was no significant relationship in Japan (*χ* ^2^ = 1.51, *P* = 0.22; likelihood ratio test). The size of each plot is proportional to the number of data points it represents.

On the other hand, the density of *B. chinensis* nests was highly negatively associated with nest density of log-nesting native ant species in the introduced range (*χ* ^2^ = 11.99, *P* < 0.001), while only marginally in the native range (*χ* ^2^ = 3.84, *P* = 0.050; Fig. 4a). Similarly to previous studies^10,17,18^, the presence of *B. chinensis* nests was strongly associated with lower native ant diversity in the introduced range (*χ* ^2^ = 29.68, *P* < 0.0001) but not within its native range (*χ* ^2^ = 1.51, *P* = 0.22; likelihood ratio test; Table 1, Fig. 4b). The low species diversity observed in areas invaded by *B. chinensis*, has previously been hypothesized as a consequence of *B. chinensis* increased predation on ants in its introduced range in comparison to its native range^10^. Our results, however, suggest that this scenario is unlikely in as much as we observed no differences between the δ^15^N and δ^13^C values of *B. chinensis* between native and introduced ranges (which would be expected if *B. chinensis* were feeding on ants since ants occupy a higher trophic level than do termites). We suggest instead that the strong effect of *B. chinensis* on native ants is more likely due to the ability of *B. chinensis* to compete for (and draw down) resources resulting from its expanded dietary breadth.

**Table 1:** Frequency of ant species in sites with and without *B. chinensis* in the native and introduced ranges of this species.

Native range			Introduced range		
species	with *B.c*.	no *B.c*.	species	with *B.c*.	no *B.c*.
***Brachyponera***					
*B. chinensis*	1.000	0.000	*B. chinensis*	1.000	0.000
*B. nakasujii*	0.000	0.026			
***Aphaenogaster***					
			*A. carolinensis*	0.008	0.111
			*A. fulva*	0.015	0.060
			*A. lamellidens*	0.008	0.033
			*A. rudis*	0.000	0.008
***Camponotus***					
*C. bishamon*	0.000	0.003	*C. castaneus*	0.008	0.003
*C. devestivus*	0.000	0.002	*C. chromaiodes*	0.068	0.030
*C. itoi*	0.000	0.006	*C. nearcticus*	0.000	0.014
*C. japonicus*	0.000	0.002	*C. snellingi*	0.000	0.003
*C. kiusiuensis*	0.000	0.026	*Camponotus sp.C*	0.000	0.003
*C. obscuripes*	0.000	0.021	*Camponotus sp.D*	0.000	0.003
*C. quadrinotatus*	0.000	0.003	*Camponotus sp.E*	0.000	0.003
*C. vitiosus*	0.000	0.011			
*C. yamaokai*	0.000	0.008			
***Crematogaster***					
*C. matsumurai*	0.000	0.005	*C. ashmeadi*	0.000	0.011
*C. teranishii*	0.000	0.030	*C. lineolata*	0.000	0.022
			*C. pilosa*	0.000	0.011
***Cryptopone***					
*C. sauteri*	0.021	0.026	*C. gilva*	0.008	0.000
***Euponera***					
*E. pilosior*	0.000	0.008			
***Formica***					
			*F. subsericea*	0.015	0.016
***Hypoponera***					
*H. beppin*	0.021	0.002	*H. opacior*	0.000	0.008
*H. sauteri*	0.021	0.000			
***Lasius***					
*L. alienus*	0.000	0.002	*L. alienus*	0.023	0.084
*L. flavus*	0.000	0.002	*L. flavus*	0.015	0.019
*L. hayashi*	0.000	0.003	*L. umbratus*	0.000	0.005
*L. japonicus*	0.021	0.006	*Lasius sp.(A)*	0.000	0.003
*L. productus*	0.083	0.044	*Lasius sp.(B)*	0.000	0.003
*L. nipponensis*	0.000	0.003			
***Monomorium***					
*M. intrudens*	0.000	0.002	*M. minimum*	0.045	0.087
*M. triviale*	0.000	0.002			
***Myrmica***					
			*Myrmica sp*.	0.000	0.003
***Nylanderia***					
*N. flavipes*	0.000	0.014	*N. faisonensis*	0.000	0.008
***Ochetellus***					
*O. glaber*	0.000	0.008			
***Pheidole***					
*Ph. noda*	0.000	0.005	*Ph. bicarinata*	0.000	0.003
			*Ph. dentata*	0.000	0.011
***Polyrhachis***					
*P. lamellidens*	0.021	0.000			
***Ponera***					
			*P. pennsylvanica*	0.000	0.008
***Pristomyrmex***					
*P. punctatus*	0.000	0.026			
***Solenopsis***					
			*S. invicta*	0.000	0.003
			*S. molesta*	0.008	0.060
***Stenamma***					
*S. owstoni*	0.000	0.002			
***Strumigenys***					
*S. lewisi*	0.000	0.009			
***Tapinoma***					
			*T. sessile*	0.000	0.005
***Temnothorax***					
*T. congruus*	0.021	0.000	*T. curvispinosus*	0.000	0.005
*T. makora*	0.000	0.002	*T. tuscaloosae*	0.000	0.005
***Tetramorium***					
*T. tsushimae*	0.000	0.008			
***Vollenhovia***					
*V. emeryi*	0.021	0.042			
# logs sampled	48	662		132	369
# species	9	32		12	32

Overall, we demonstrate a marked expansion in feeding habits of an introduced ant. Even such a seemingly subtle dietary change in new environments can cause large impacts on the communities of introduced regions, impacts that cannot be easily predicted from the feeding habits in the native range.

## Methods

### Study sites

To compare colony density and feeding habits of *B. chinensis* between the native range (Japan) and the introduced range (United States), we conducted transect sampling in Okayama in Western Japan and in Raleigh in the United States. The climate of Raleigh is very similar to that of Okayama with mean annual precipitation around 1100 mm. The temperature in Raleigh ranges from mean annual minimum of −1 °C to mean annual maximum of 32 °C, and that in Okayama ranges from mean annual minimum of 1 °C to mean annual maximum of 33 °C.

In the native range, we conducted transect sampling at seven sites (T1-T7) in six secondary forests (Kasaiyama (T1: N34.718900, E133.934233), Washuzan (T2: N34.435047, E133.816467), Hattantoge (T3: N34.691147, E134.184311), Shimotakada (T4: N34.764981, E133.833881), Takahashi (T5: N34.795536, E133.576878), Ryuten (T6: N34.894858, E134.006953, T7: N34.893608, E134.001953)) in Okayama, Western Japan between 10am and 4pm (Fig. 1). These forests are dominated by Japanese red pine *Pinus densiflora* and *Quercus serrata* oak. One transect was run in each forest except for Ryuten where we ran two transects. The minimum distance between any two neighboring forests was 10 km.

In the introduced range, we conducted transect sampling at five sites (T8-T12) in four mature mesic deciduous forests (Schenck Memorial Forest (T8: N35.816322, W78.720964), Yates Mill Pond forest (T9: N35.721522, W78.689622), Lake Wheeler forest (T10: N35.693786, W78.698669) and Lake Johnson forest (T11: N35.762825, W78.716164, T12: N35.758286, W78.716264)) in Raleigh, North Carolina, USA between 10 am and 4 pm (Fig. 1). One transect was run in each forest except two transects were run in the Lake Johnson forest. The minimum distance between any two neighbouring forests was 3 km. This area is currently near the centre of the *B. chinensis* invaded range^10,35^.

### Transect sampling

Ant and termite sampling was conducted along seven 900-m line transects in Japan (T1-T7) and five transects in the United States (T8-T12). Along each transect we sampled 10 points, one every 100 m. Sampling was conducted in a 10-m radius around each sampling point by six trained people. In order to standardize sampling effort, ten dead logs were randomly chosen in each circle. All termites and ants were carefully extracted from logs by using a hand axe, a knife and an aspirator (Dataset S3 and S4). We defined a *B. chinensis* nest as an aggregation of more than 10 individuals in the logs and excluded the individuals walking on the surface of the logs from nest counts. Litter was also sampled from the forest floor as the baseline of δ^15^N, δ^13^C, and ∆^14^C values at each sampling site. For stable isotope and radiocarbon analyses, samples were oven-dried at 60 °C for 24 h. The termites and the ants were also placed in vials filled with 95% ethanol for species identification.

### Stable isotope analyses

For stable C and N isotope analyses, the samples (approximately 0.5 mg: a few individuals of termites and ants from each colony) were placed in folded tin capsules. Stable C and N isotope ratios were measured using a mass spectrometer (Delta^plus^ XP, Thermo Electron, Erlangen, Germany) coupled with an elemental analyser (Flash EA 1112, Thermo Electron, Erlangen, Germany). The precision of the on-line procedure was better than ± 0.2‰ for both isotope ratios. Natural abundances of ^13^C and ^15^N are expressed in per mil (‰) deviation from international standards: δ^13^C or δ^15^N = (*R*_sample_/*R*_standard_ − 1) × 1000, where *R* in δ^13^C or δ^15^N is ^13^C/^12^C or ^15^N/^14^N, respectively. Pee Dee Belemnite and atmospheric nitrogen were used as the international standards for carbon and nitrogen, respectively.

### Radio isotope analyses and diet age estimation

For radiocarbon analysis, the samples (estimated to contain approximately 1 mg C: several individuals of termites and ants from each colony) were combusted in evacuated and sealed Vycor tubes with CuO and Ag wire at 850 °C for 2h. After cooling, the Vycor tubes were cracked on a vacuum line, and the CO_2_ was cryogenically purified. The purified CO_2_ was graphitized under Fe catalysis at 650 °C for 4.5h^36^. The graphite samples were sent to Rafter Radiocarbon Laboratory, Institute of Geological and Nuclear Sciences, New Zealand, for accelerator mass spectrometry measurements of radiocarbon. Radiocarbon values were reported as ∆^14^C (‰), which is the part per thousand deviations from the activity of 19th century wood and were corrected for fractionation using stable C isotope ratios of the samples^37,38^. The average analytical error was ± 3.7‰.

We estimated the diet age by calculating the difference between the sample collection year and the year (t) when the ∆^14^C value of a sample matched that of atmospheric CO_2,_ as described previously by Hyodo *et al*.^27^. The year (t) was calculated using the regression curve, year (t) = 2074 − 16.71 ln(∆^14^C) (R^2^ = 0.997, *P* < 0.0001), which is based on ^14^CO_2_ data for the northern hemisphere from 1977 to 1999^38^. Because the deciduous leaf litter were collected both in Japan and United States, the ages of litter was expected to be 0 years. However, our results showed that the leaf litter samples collected both in Japan and United States showed negative ages (Dataset S1) likely due to the effect of fossil fuel burning on atmospheric ^14^CO_2_^39,40^. Therefore, we corrected the diet ages of ants and termites for the fossil fuel effects by adding years so that leaf litter samples had ages of 0 years old. The analytical error of ∆^14^C indicated that the diet age could be estimated with a precision of 1−2 years. The time elapsed since the production of the litter was also estimated as diet age for comparison.

### Statistical Analyses

To explore differences in diet ages of ants, termites, and litter between Japan and United States, we applied a generalized linear mixed model (GLMM) in which the diet ages of ants, termites, and litter were treated as response variables, the study sites (native and introduced ranges), the sample types (i.e., ants, termites, and litter), and the interaction term (i.e., the study sites × the sample types) as explanatory variables, and the sampling points in Japan and United States as a random effect. We also used GLMM to examine differences in feeding habits between the native and introduced ranges using stable C and N isotopes. In this model, the δ^13^C and δ^15^N of ants, termites, and litter were treated as response variables, the study sites (native and introduced ranges), the sample types (ants, termites, and litter), and the interaction term (the study sites × the sample types) as explanatory variables, and the sampling locations as a random effect. A post hoc test (Tukey-Kramer HSD test) was applied to examine the overall significant difference at *P* = 0.05.

We used a GLMM with binomial errors in which the proportion of logs (out of 10) with termites in a sampling site was treated as a response variable, and GLMM with Poisson errors in which the number of ant species (except *B. chinensis*) in a sampling site was treated as a response variable. In these models, the proportion of logs with *B. chinensi*s colonies in a sampling point was treated as an explanatory variable, and the transect as a random effect. We used GLMM with binomial errors in which termite presence/absence in a log was treated as a response variable, the presence/absence of *B. chinensis* in the log as an explanatory variable, and the sampling site nested in the transect as a random effect.

## Electronic supplementary material

Supplementary Information

Dataset S1

Dataset S2

Dataset S3

Dataset S4
