# Supplementary material for: Radiocarbon analysis reveals expanded diet breadth associates with the invasion of a predatory ant
Source: Sci Rep. 2017 Nov 3;7:15016. doi: 10.1038/s41598-017-15105-1 (PMC5670172; doi:10.1038/s41598-017-15105-1)
Supplement: Supplementary file 1 — Supplementary Information [file 41598_2017_15105_MOESM1_ESM.doc]

Supplementary information

**Radiocarbon analysis reveals expanded diet breadth associates with the invasion of a predatory ant**

Wataru Suehiro^1^*, Fujio Hyodo^2^*, Hiroshi O. Tanaka^2^, Chihiro Himuro^3^, Tomoyuki Yokoi^4^, Shigeto Dobata^1^, Benoit Guénard^5^, Robert R. Dunn^6^, Edward L. Vargo^7^, Kazuki Tsuji^8^, Kenji Matsuura^1†^

**Table S1** Number of the nests of termite and ant species collected by each transect sampling in Japan

|  | Transect ID | | | | | | |
| --- | --- | --- | --- | --- | --- | --- | --- |
|  | T1 | T2 | T3 | T4 | T5 | T6 | T7 |
| **Termites** |  |  |  |  |  |  |  |
| *Reticulitermes speratus* | 11 | 0 | 15 | 15 | 12 | 8 | 12 |
| **Ants** |  |  |  |  |  |  |  |
| *Brachyponera chinensis* | 7 | 16 | 4 | 3 | 0 | 8 | 3 |
| *Brachyponera nakasujii* | 1 | 3 | 1 | 4 | 0 | 5 | 0 |
| *Camponotus bishamon* | 0 | 0 | 1 | 0 | 0 | 0 | 1 |
| *Camponotus devestivus* | 0 | 0 | 0 | 0 | 0 | 0 | 1 |
| *Camponotus itoi* | 0 | 0 | 3 | 0 | 0 | 1 | 0 |
| *Camponotus japonicus* | 0 | 0 | 0 | 1 | 0 | 0 | 0 |
| *Camponotus kiusiuensis* | 3 | 0 | 9 | 0 | 1 | 3 | 1 |
| *Camponotus obscuripes* | 0 | 0 | 0 | 3 | 3 | 1 | 7 |
| *Camponotus quadrinotatus* | 0 | 0 | 1 | 1 | 0 | 0 | 0 |
| *Camponotus vitiosus* | 0 | 5 | 1 | 0 | 0 | 1 | 0 |
| *Camponotus yamaokai* | 0 | 0 | 1 | 0 | 0 | 2 | 2 |
| *Crematogaster matsumurai* | 0 | 0 | 0 | 1 | 0 | 0 | 2 |
| *Crematogaster teranishii* | 9 | 1 | 1 | 4 | 4 | 0 | 1 |
| *Cryptopone sauteri* | 2 | 0 | 1 | 4 | 7 | 3 | 1 |
| *Euponera pilosior* | 0 | 0 | 0 | 0 | 0 | 1 | 4 |
| *Hypoponera beppin* | 0 | 0 | 1 | 1 | 0 | 0 | 0 |
| *Hypoponera sauteri* | 0 | 0 | 0 | 0 | 0 | 1 | 0 |
| *Lasius alienus* | 0 | 0 | 0 | 0 | 0 | 1 | 0 |
| *Lasius flavus* | 0 | 0 | 0 | 0 | 0 | 0 | 1 |
| *Lasius hayashi* | 0 | 0 | 1 | 0 | 0 | 0 | 1 |
| *Lasius japonicus* | 3 | 0 | 1 | 0 | 0 | 1 | 0 |
| *Lasius productus* | 5 | 1 | 4 | 11 | 4 | 5 | 3 |
| *Lasius nipponensis* | 0 | 0 | 2 | 0 | 0 | 0 | 0 |
| *Monomorium intrudens* | 0 | 0 | 0 | 1 | 0 | 0 | 0 |
| *Monomorium triviale* | 0 | 0 | 0 | 0 | 1 | 0 | 0 |
| *Nylanderia flavipes* | 0 | 1 | 0 | 0 | 3 | 2 | 3 |
| *Ochetellus glaber* | 0 | 1 | 4 | 0 | 0 | 0 | 0 |
| *Pheidole noda* | 0 | 3 | 0 | 0 | 0 | 0 | 0 |
| *Polyrhachis lamellidens* | 1 | 0 | 0 | 0 | 0 | 0 | 0 |
| *Pristomyrmex punctatus* | 4 | 0 | 5 | 0 | 5 | 0 | 3 |
| *Stenamma owstoni* | 0 | 0 | 1 | 0 | 0 | 0 | 0 |
| *Strumigenys lewisi* | 1 | 0 | 2 | 1 | 0 | 0 | 2 |
| *Temnothorax congruus* | 0 | 0 | 0 | 0 | 0 | 0 | 1 |
| *Temnothorax makora* | 0 | 0 | 0 | 0 | 0 | 0 | 1 |
| *Tetramorium tsushimae* | 0 | 0 | 3 | 1 | 1 | 0 | 0 |
| *Vollenhovia emeryi* | 10 | 3 | 2 | 4 | 0 | 6 | 4 |

**Table S2** Number of the nests of termite and ant species collected by each transect sampling the United States

|  | Transect ID | | | | |
| --- | --- | --- | --- | --- | --- |
|  | T8 | T9 | T10 | T11 | T12 |
| **Termites** |  |  |  |  |  |
| *Reticulitermes flavipes* | 30 | 27 | 13 | 17 | 34 |
| *Reticulitermes virginicus* | 9 | 10 | 15 | 9 | 8 |
| *Reticulitermes hageni* | 0 | 0 | 1 | 0 | 0 |
| **Ants** |  |  |  |  |  |
| *Brachyponera chinensis* | 29 | 4 | 25 | 25 | 48 |
| *Aphaenogaster carolinensis* | 8 | 12 | 3 | 9 | 10 |
| *Aphaenogaster fulva* | 1 | 12 | 1 | 7 | 3 |
| *Aphaenogaster lamellidens* | 0 | 3 | 3 | 3 | 4 |
| *Aphaenogaster rudis* | 1 | 0 | 0 | 1 | 1 |
| *Camponotus castaneuis* | 0 | 1 | 0 | 0 | 1 |
| *Camponotus chromaiodes* | 7 | 5 | 2 | 5 | 1 |
| *Camponotus nearcticus* | 1 | 0 | 4 | 0 | 0 |
| *Camponotus snellingi* | 0 | 0 | 0 | 1 | 0 |
| *Camponotus sp.(A)* | 1 | 0 | 0 | 0 | 0 |
| *Camponotus sp.(B)* | 1 | 0 | 0 | 0 | 0 |
| *Camponotus sp.(C)* | 1 | 0 | 0 | 0 | 0 |
| *Crematogaster ashmeadi* | 1 | 1 | 2 | 0 | 0 |
| *Crematogaster lineolata* | 3 | 3 | 1 | 0 | 1 |
| *Crematogaster pilosa* | 0 | 0 | 4 | 0 | 0 |
| *Cryptopone gilva* | 0 | 0 | 0 | 1 | 0 |
| *Formica subsericea* | 1 | 1 | 0 | 3 | 3 |
| *Hypoponera opacior* | 1 | 1 | 0 | 1 | 0 |
| *Lasius alienus* | 5 | 13 | 4 | 6 | 6 |
| *Lasius flavus* | 0 | 0 | 0 | 9 | 0 |
| *Lasius umbratus* | 0 | 1 | 0 | 1 | 0 |
| *Lasius sp.(A)* | 0 | 0 | 0 | 1 | 0 |
| *Lasius sp.(B)* | 0 | 0 | 0 | 1 | 0 |
| *Monomorium minimum* | 6 | 9 | 22 | 1 | 0 |
| *Myrmica sp.* | 0 | 0 | 0 | 1 | 0 |
| *Nylanderia faisonensis* | 0 | 2 | 0 | 1 | 0 |
| *Pheidole bicarinata* | 1 | 0 | 0 | 0 | 0 |
| *Pheidole dentata* | 2 | 0 | 2 | 0 | 0 |
| *Ponera pennsylvanica* | 0 | 2 | 0 | 0 | 1 |
| *Solenopsis invicta* | 0 | 0 | 1 | 0 | 0 |
| *Solenopsis molesta* | 2 | 11 | 2 | 7 | 1 |
| *Tapinoma sessile* | 0 | 1 | 1 | 0 | 0 |
| *Temnothorax curvispinosus* | 0 | 1 | 0 | 0 | 1 |
| *Temnothorax tuscaloosae* | 2 | 0 | 0 | 0 | 0 |
